# GutMIND: A multi-cohort machine learning framework for integrative characteristics of the microbiota-gut-brain axis in neuropsychiatric disorders

**DOI:** 10.1080/19490976.2026.2630563

**Published:** 2026-02-16

**Authors:** Yanmei Ju, Shutian Lin, Shaohua Hu, Xin Jin, Liang Xiao, Tao Zhang, Yudan Zhang, Liping Zhang, Xiancang Ma, Feng Zhu, Ruijin Guo

**Affiliations:** a College of Life Sciences, University of Chinese Academy of Sciences, Beijing, People's Republic of China; b BGI Research, Wuhan, People's Republic of China; c BGI Research, Shenzhen, People's Republic of China; d State Key Laboratory of Genome and Multi-omics Technologies, BGI Research, Shenzhen, People's Republic of China; e BGI Precision Nutrition, Shenzhen, People's Republic of China; f Department of Psychiatry, First Affiliated Hospital, Zhejiang University School of Medicine, Hangzhou, People's Republic of China; g The Key Laboratory of Mental Disorder’s Management of Zhejiang Province, Hangzhou, People's Republic of China; h Shenzhen Engineering Laboratory of Detection and Intervention of Human Intestinal Microbiome, BGI Research, Shenzhen, People's Republic of China; i Qingdao-Europe Advanced Institute for Life Sciences, BGI Research, Qingdao, People's Republic of China; j Shaan Probiomicros Co. Ltd., Suzhou, People's Republic of China; k Ningbo mBioU Biopharma Co. Ltd., Ningbo, People's Republic of China; l Shaanxi Provincial Key Laboratory of Biological Psychiatry, Xi’an, People's Republic of China; m Department of Psychiatry, the First Affiliated Hospital of Xi’an Jiaotong University, Xi'an, People's Republic of China; n Center for Translational Medicine, the First Affiliated Hospital of Xi’an Jiaotong University, Xi'an, People's Republic of China

**Keywords:** Microbiota-gut-brain axis, neuropsychiatric disorders, machine learning, microbial biomarkers

## Abstract

Emerging evidence underscores bidirectional communication along the microbiota-gut-brain axis in neuropsychiatric disorders. However, the field lacks dedicated metagenomic resources with standardized phenotyping for these conditions. Existing single-cohort studies face inherent limitations due to restricted sample sizes, confounding heterogeneity, and methodological fragmentation, compromising reproducibility and mechanistic insights. To overcome these challenges, we constructed the Gut Microbiome in Multinational Integrated Neuropsychiatric Disorders (GutMIND) database, a comprehensive resource integrating shotgun metagenomic data with harmonized metadata. Adhering to a standardized preprocessing protocol and rigorous quality control workflow, this dataset represents the largest gut-brain microbiome repository to date, encompassing 31 studies across 12 countries (*n* = 3,492) spanning 14 neuropsychiatric conditions. Utilizing this dataset, we characterized microbial community heterogeneity, which was significantly elevated in patients compared to healthy controls. Subsequently, we developed a computational framework, MetaClassifier, enabling the diagnosis of neuropsychiatric disorders and the identification of microbial biomarkers. Employing a comprehensive two-stage validation strategy, we first assessed the model utilizing taxonomic abundance profiles via nested cross-validation in the high-quality discovery cohort (*n* = 2,734), achieving a mean AUROC of 0.69 (range: 0.55–0.78) across 8 disorders. Its robustness was further confirmed in an independent platform-extended validation cohort (*n* = 400), yielding a mean AUROC of 0.71 (range: 0.60–0.76). We also developed the Microbial Gut-Brain Axis Health Index (MGBA-HI), which effectively distinguished neuropsychiatric status in both the high-quality cohort and the platform-extended cohort. Furthermore, integrative analysis of health-abundant species, index-derived biomarkers, and ecological prevalence, we identified 9 core neuropsychiatric-protective microbiota. These species predominantly exhibited metabolic capacities linked to glutamate synthesis and acetate production. Building upon this, the GutMIND framework ensures robust cross-cohort comparability while minimizing technical heterogeneity, thereby enhancing inferential rigor in gut microbiome-neuropsychiatry research. Notably, the MetaClassifier, MGBA-HI, and core microbiota hold translational potential for developing microbiome-based prognostic tools and personalized therapeutic strategies in neuropsychiatric disorders. The source code and usage instructions for MetaClassifier are accessible at https://github.com/juyanmei/MetaClassifier.

## Introduction

The microbiota-gut-brain axis (MGBA) has emerged as a paradigm-shifting framework for elucidating bidirectional communication between the gut microbiota and central nervous system (CNS) function. Accumulating evidence underscores that gut microbiota regulates neurodevelopment and behavior through immunomodulatory pathways (e.g., cytokine signaling), microbial metabolite production (e.g., short-chain fatty acids, SCFAs), and direct modulation of neurotransmitter systems.^1^ Clinical observations reveal distinct taxonomic and functional signatures within the gut microbiota, highlighting their role in a broad spectrum of neuropsychiatric disorders. These conditions span traditional clinical boundaries and include developmental disorders like autism spectrum disorder (ASD),^2-9^ psychiatric conditions such as major depressive disorder (MDD)^10-13^ and schizophrenia (SCZ)^14-19^ and neurodegenerative diseases with psychiatric features Parkinson’s disease (PD).^20-28^ For instance, dysregulated gut microbiota in PD exhibits reduced abundance of SCFA-producing species,^29^ correlating with behavioral deficits and neurotransmitter imbalances (e.g., GABA dysregulation mediated by Bacteroides genera).^30^ Similarly, our previous study demonstrated that schizophrenia is associated with functional dysbiosis in gut microbial communities, characterized by the disruption of short-chain fatty acid biosynthesis and neurotransmitter metabolism.^19^ Beyond disease associations, longitudinal metagenomic studies reveal that gut microbial community stability and resilience, hallmarks of a healthy microbiome, are compromised in neurodegenerative conditions.^31^ While fecal microbiota transplantation (FMT) or prebiotic/probiotic interventions demonstrate therapeutic potential by restoring microbial diversity and metabolic potential, interindividual variability in treatment response highlights the need for personalized therapeutic strategies.^32^

Advancements in metagenomic sequencing technologies and the accumulation of MGBA-associated datasets have illuminated critical translational bottlenecks in clinical applications. Existing databases exhibit pronounced gaps in global representation for neuropsychiatric disorders. Existing repositories, including American Gut,^33^ curatedMetagenomics,^34^ and GMrepo,^35^^,^^36^ suffer from geographical biases and insufficient coverage of neuropsychiatric conditions. While PsycGM^37^ provides psychiatric-focused data, its limited coverage of metagenomic profiles represents a critical gap in microbial characterization. Such limitations exacerbate challenges posed by sample heterogeneity, including small cohort sizes, restricted demographic diversity, and non-standardized sampling protocols, which collectively skew gut microbiota compositional analyzes and hinder cross-population generalizability. Compounding these issues, methodological fragmentation arises from divergent bioinformatics pipelines (e.g., differing normalization strategies, taxonomic classifiers) and inconsistent reference databases, leading to conflicting microbial signatures even within overlapping neuropsychiatric phenotypes. In response, recent studies have successfully employed large-scale meta-analyzes and machine learning to identify robust markers in specific disorders, such as PD^38^ and SCZ.^39^ However, these efforts remain largely siloed within single-disease contexts. Current indices like the Gut Microbiome Health Index (GMHI)^40^ or Wellness Index (GMWI)^41^ are insufficient here, as they were optimized for general metabolic health and lack the sensitivity to capture shared neuroactive signatures across neuropsychiatric conditions. Notably, the absence of a consensus “healthy” microbiome framework further complicates efforts to link microbial dynamics to neurobiological outcomes.

To address these gaps, we established the GutMIND database, a standardized multi-cohort resource integrating large-scale, shotgun metagenomic data with structured metadata. Specifically, we hypothesized that shared microbial signatures exist across neuropsychiatric conditions, and that a unified index derived from them would outperform traditional diversity metrics. Utilizing a unified bioinformatics pipeline, we systematically characterize pan-microbiome features and identify disease-specific differential microbial signatures. Based on these, we developed MetaClassifier: a generalized machine-learning framework, capable of high-performance neuropsychiatric disorder diagnosis and identification of transdiagnostic microbial biomarkers. Additionally, we construct the Microbial Gut-Brain Axis Health Index (MGBA-HI) to reflect the overall microbial signature that distinguishes the neuropsychiatric and disease state from controls. Moreover, through integrative analysis of dysregulated species and classifier-derived biomarkers, we pinpointed core protective microbiota implicated in neuropsychiatric resilience. Consequently, GutMIND framework will inform precision-targeted therapies, bridging bench-to-bedside translational divides in neuropsychiatric disorders through systematic interrogation of the microbiota-gut-brain axis.

## Materials and methods

### Study collection

We conducted a search for the keywords “gut microbiota”, “shotgun sequencing”, “gut-brain axis”, “mental disease”, among others, in PubMed and Google Scholar (as of June 2024). A primary study exclusion criterion: (1) corresponding metagenomics data available for download from the National Center for Biotechnology Information (NCBI) and China National GeneBank (CNGB) databases. (2) relevant disease information. Metadata were extracted through manual curation of publications, database records, and author communications. The GutMIND database comprised 34 datasets derived from 31 independent studies, stratified by disease type and sequencing approach (details shown in Table S1).

### Sequencing data processing, taxonomic and functional profiling

Raw sequence files from NCBI/CNGB were processed using a bioinformatic pipeline. First, fastp^42^^,^^43^ (v0.23.2) was used for quality control to remove low-quality reads and bases. Subsequently, Bowtie2^44^ (v2.5.3) was employed for host sequence removal against the human reference genome (GRCh38). The resulting clean reads were then analyzed for taxonomic profiling using MetaPhlAn4^45^ (v4.0.6) with the mpa_vOct22_CHOCOPhlAnSGB_202212 database, and for functional pathway analysis using HUMAnN^46^ (v3.9).

### Sample filtering and species removal

We applied a series of stringent filters to ensure the quality and relevance of our dataset. Specifically, we excluded any sample with a read count below 10 million to ensure sufficient sequencing depth and minimize technical variation.^47^ Additionally, samples with more than 30% unclassified sequences at the species level were removed to effectively exclude a small number of potential low-quality outlier samples, while retaining over 95% of the dataset for the final meta-analysis. We also excluded samples from individuals with a BMI greater than 30 kg/m^2^ to reduce the confounding influence of obesity on the microbiota-gut-brain axis.^48^ Furthermore, if a study had fewer than 10 samples remaining after these filters, the entire study was discarded. After implementing these comprehensive filters, a total of 3,492 samples remained in our final dataset. To minimize technical and phenotypic heterogeneity across studies, we categorized the database into three groups: the high-quality dataset comprising Illumina paired-end sequenced samples with matched case-control designs (8 disease types), the platform-expanded dataset containing non-Illumina samples covering 3/8 overlapping diseases for platform comparison, and the disease-expanded dataset including samples representing additional disease phenotypes beyond the core set, enabling robust cross-platform validation while maximizing disease coverage (details shown in Table S1). Species profiling was refined using a relative abundance cutoff of 1 × 10^−4^ and an occurrence rate threshold of 1%. Specifically, samples with missing metadata were coded as “NA” and removed for statistical analyzes. The applied abundance and occurrence thresholds, along with sample inclusion criteria, reduce stochastic noise, minimize inter-sample heterogeneity, and improve the robustness and reproducibility of downstream analyzes, including differential abundance and predictive modeling.

### Batch effects correlation

Batch effect correction was applied to each disease cohort using three distinct methods: MMUPHin^49^ (adjust_batch function from R package MMUPHin v1.18.1), DEBIAS-M^50^ (DebiasMClassifier from Python module debiasm v0.0.1), and ComBat^51^ (ComBat function from R package sva (v3.52.0)), all run with default parameters while explicitly including disease status as a covariate to preserve the biological signal of interest. These methods were selected as they represent complementary strategies that respectively address global linear shifts, compositional and group-dependent effects, and signal–batch confounding. ComBat is a classical empirical Bayes method that models batch effects as linear additive shifts, providing a conventional baseline correction. MMUPHin extends this framework to microbiome compositional data, incorporating group structure and covariate effects to better handle compositional constraints. DEBIAS-M, in contrast, employs a debiased representation learning framework that removes batch effects confounded with biological signals. Principal Coordinates Analysis (PCoA) plots based on Bray-Curtis distances were generated to visualize microbial community distributions before and after batch correction. Comparative analyzes included pre-correction and post-correction results for each method across all disease cohorts.

### PERMANOVA

The effectiveness of correction was quantitatively evaluated by calculating R² values using PERMANOVA (Permutational Multivariate Analysis of Variance) (999 permutations). PERMANOVA was conducted using the adonis2 function in the vegan^52^ (v2.7.1) R package, with Bray-Curtis distance as input. The R^2^ values represent the proportion of variance explained by the tested factor (e.g., disease status or batch effect, depending on the analysis).

### Diversity

Alpha diversity was calculated using the Shannon index by vegan. Beta diversity was evaluated by Bray-Curtis dissimilarity and Jaccard index using vegan.^52^

### Model construction and evaluation based on MetaClassifier

#### Base model

MetaClassifier was implemented using Python's scikit-learn (v1.7.2) library^53^ in Python v3.11. The framework systematically evaluates a suite of candidate classification algorithms, including linear discriminators (Elastic Net, Lasso-regularized logistic regression, logistic regression) and tree-based ensembles (CatBoost v1.2.7,^54^ Random Forest). Linear models were chosen for their interpretability and effectiveness in high-dimensional data. Random Forest is an ensemble tree-based method that captures non-linear relationships and interactions among features. CatBoost is a gradient boosting algorithm that efficiently handles categorical variables and complex feature interactions. Feature importance was quantified based on the model type. For Lasso, ElasticNet, and Logistic Regression, the absolute values of model coefficients were used. For Random Forest, Gini importance (Mean Decrease in Impurity, MDI) was used. For CatBoost, the model provided PredictionValuesChange metric, which quantifies the average effect of each feature on model predictions. The final model was selected from these candidates based on the performance of each algorithm, primarily the nested cross-validation (CV) Area Under the Receiver Operating Characteristic Curve (AUROC) and, where applicable, the Leave-One-Cohort-Out (LOCO) CV AUROC.

#### Final model construction

To obtain a robust and unbiased estimate of model performance, we employed a 10-times repeated, 5-fold nested CV strategy. In this procedure, an outer 5-fold loop was used for model evaluation, while an inner 5-fold loop was dedicated to feature selection and Bayesian hyperparameter optimization (Catboost: depth [4, 12], learning_rate [0.01, 0.3], iterations [100, 1000], and l2_leaf_reg [1, 20]). To prevent information leakage, both feature selection and hyperparameter tuning were performed strictly within the inner loops of the nested cross-validation structure, ensuring an unbiased evaluation. We reported the mean AUROC from the outer loop's test folds as the nested CV AUROC, which serves as an unbiased estimate of generalization performance. For diseases with data from three or more cohorts, we implemented a LOCO CV scheme as the outer loop to assess the model's generalizability across different study populations, while the inner 5-fold loop was maintained for feature selection and hyperparameter tuning. Depending on the selected features, we employed a 5-fold CV and used the full data to do Bayesian hyperparameter optimization and reported this tuning AUROC. Finally, we constructed the final model and reported the final fit AUROC using the full data. This final model was then subjected to further evaluation as described below. All related code and the parameter ranges used in this study have been made publicly available on GitHub at: https://github.com/juyanmei/MetaClassifier.

#### Final model evaluation

We constructed two distinct sets of models: ‘Single-Study Model’, each built using data from an individual study, and ‘Single-Disease Model’, built by integrating all relevant studies for a given disease. To rigorously evaluate the performance of our constructed models, we implemented a multi-tiered validation strategy designed to assess three key properties: generalization, robustness, and specificity.

Cross-Study Generalization: This strategy was applied to models built using data from a single study (single-study model). To assess its generalization ability, a model trained on one study was tested on data from all other available studies of the same disease.

Robustness Assessment: Robustness was primarily assessed for our single-disease models which were trained on pooled data from multiple studies. Whenever a suitable independent dataset was available, our final integrated disease model was tested on this external data. This serves as a gold-standard measure of robustness, demonstrating the model's performance on data completely unseen during any training or optimization phase.

Cross-Disease Specificity: To ensure our models were learning disease-specific biological signals rather than general artifacts, we performed cross-disease validation. A final model trained to classify a specific disease was applied to datasets from entirely different diseases. High predictive performance on its target disease but poor performance on other diseases demonstrates the model's specificity.

#### MGBA-HI calculation

To calculate the MGBA-HI (Microbial Gut-Brain Axis Health Index) for neuropsychiatric disorder risk assessment, we first developed a disease classification model using CatBoost algorithm with LOCO CV. This model was trained on DEBIAS-M corrected microbial abundance profiles derived from the entire high-quality dataset. The optimal classification threshold was determined by maximizing Youden's Index (Sensitivity + Specificity-1). MGBA-HI was calculated by 0 - (probability of disease - optimal classification threshold). This formulation centers the index at the decision boundary (0), such that positive values intuitively represent a ‘healthy’ state (probability < optimal classification threshold) and negative values represent a ‘disease’ state, quantifying the confidence of the health classification. Since our data were obtained using MetaPhlAn4^45^ with mpa_vOct22_CHOCOPhlAnSGB_202212, we did not compare them with the GMHI (Gut Microbiome Health Index)^40^ or GMWI2 (Gut Microbiome Wellness Index 2),^41^ which are based on MetaPhlAn2 and MetaPhlAn3 outputs, respectively. Differences in marker sets and abundance estimation between MetaPhlAn versions prevent direct calculation or comparison of these indices.

#### GBM detection

Through MetaPhlAn4 with mpa_vOct22_CHOCOPhlAnSGB_202212 genome database tracing, we identified 21 species-level genome bins corresponding to the 9 core neuropsycho-protective microbiota. Gene functional annotation of 21 genomes was performed using EggNOG (v5.1)^55^ to identify conserved protein domains. GBM coverage was calculated via Omixer-RPM (version 1.1; https://github.com/raeslab/omixer-rpm). A GBM was classified as present in a microbial genome if it achieved ≥ 66% coverage and it was considered present if detected in ≥1 representative genome at the species level.^56^

#### Statistical analysis

Comparison analysis across-cohort was using MaAsLin3,^57^ which can handle relative abundance and prevalence abundance. Multiple testing correction was performed using the Benjamini-Hochberg (BH) procedure. To synthesize the multivariable association results between microbial features and clinical metadata generated by MaAsLin3, we performed a meta-analysis. Effect sizes and their corresponding standard errors from the MaAsLin3 output were pooled using a random-effects model within the meta package (v8.2-1) in R programming language. The differences in MGBA-HI and Shannon index between patients and healthy controls were assessed using Wilcoxon rank-sum tests. Effect sizes were assessed via Cliff’s delta (Disease vs. Health), where negative values indicate lower levels in patients and positive values indicate elevation in patients. Correlation analysis between species markers and clinical phenotypes was performed using Spearman correlation. The Spearman correlation analyzes were performed using the R, specifically with the cor.test function from the base stats package. To provide robust confidence intervals for the correlation coefficients, we employed a bootstrap resampling approach with 10,000 iterations. All statistical analyzes conducted in R were performed using version 4.4.1.

## Results

### Overview of the GutMIND database and integrative multi-cohort analytical framework

**Figure 1.**
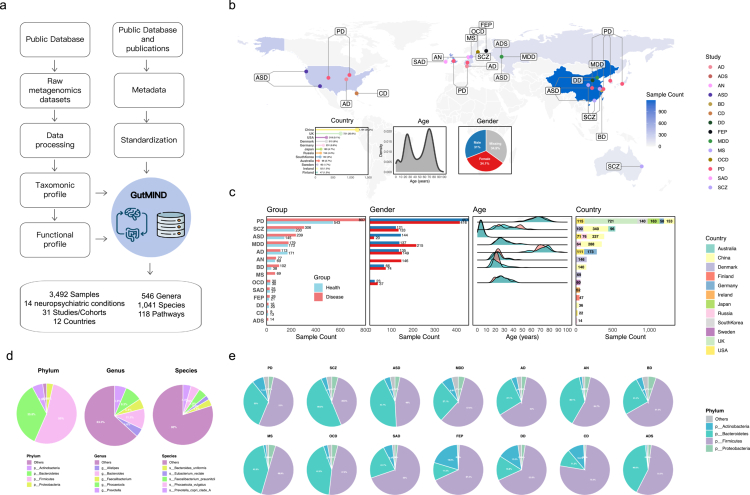
Overview of the GutMIND database. a) Workflow for the construction of the GutMIND database and core characteristics. b) Demographic characteristics of GutMIND including geographic distribution, age stratification, and gender ratio. c) Disease-specific features showing sample size, gender distribution, age range, and geographical coverage for each diagnostic category. d) Microbial composition of the GutMIND cohort across taxonomic levels (Phylum, Genus, Species). e) Phylum-level taxonomic composition across various disease cohorts.

To comprehensively delineate microbiota-neuropsychiatric associations with cross-regional validity, we conducted the Gut Microbiome in Multinational Integrated Neuropsychiatric Disorders (GutMIND) database. We acquired shotgun metagenomic data from NCBI repositories and manually curated clinical metadata from original publications. Uniform bioinformatics processing was performed on all sequencing data, followed by systematic organization and integration of the metadata. All datasets underwent standardized processing with stringent quality control (methods), MetaPhlAn4 and HUMAnN3 were used for taxonomic profiling and functional profiling (Figure 1a, Figure S1a).

The final GutMIND resource integrates 3,492 human gut microbiome samples (21.1 Terabases (Tb) of sequencing data) derived from 31 studies across 12 countries, representing a major integrated resource for gut microbiome studies in neuropsychiatric disorders. Geographically, China contributed the largest subset (*n* = 1,194; 34.2%), followed by the United Kingdom (*n* = 721; 20.6%), with remaining samples distributed across North America, Europe, Asia, and Oceania. The GutMIND cohort demonstrates broad demographic diversity, encompassing participants aged 1–95 years with balanced gender representation (1,083 males [47.6%] and 1,190 females [52.4%]) based on available phenotypic metadata (Figure 1a,b). This integrated resource covers 14 major neuropsychiatric conditions, with Parkinson's disease (PD; *n* = 1,350; 38.7%) and schizophrenia (SCZ; *n* = 536; 15.3%) representing the two largest diagnostic groups (Figure 1c, Table S1). Taxonomic profiling revealed Firmicutes as the dominant phylum (53.0 ± 19%), with Bacteroides (11.1 ± 11.1%) and Phocaeicola (9.1 ± 11.0%) representing the most abundant genera, and *Phocaeicola vulgatus* (4.9 ± 8.0%) together with *Faecalibacterium prausnitzii* (4.8 ± 5.0%) constituting the prevalent species (Figure 1d). For a specific disease, the dominant phylum is still Firmicutes (57.8 ± 9.7%) (Figure 1e). HUMAnN3-based functional analysis identified 118 conserved metabolic pathways as the core functional repertoire of the gut microbiome (Figure 1a), collectively providing multi-dimensional characterization of gut microbiome features.

Our analytical framework leverages this resource through four integrated modules: (1) Identification of cohort-stable microbial signatures using multi-modal approaches (diversity metrics, differential abundance testing, and variance partitioning); (2) Disease condition-specific differential analysis; (3) Development of the MetaClassifier workflow for constructing diagnostic models and deriving the Microbiota-Gut-Brain Axis Health Index (MGBA-HI); and (4) Systems-level analysis revealing a neuroprotective taxon consortium consistently associated with mental health outcomes, functionally enriched for neurotransmitter biosynthesis and immunomodulation pathways (Figure S1b).

### Global characteristics of gut microbiota in neuropsychiatric disorders

**Figure 2.**
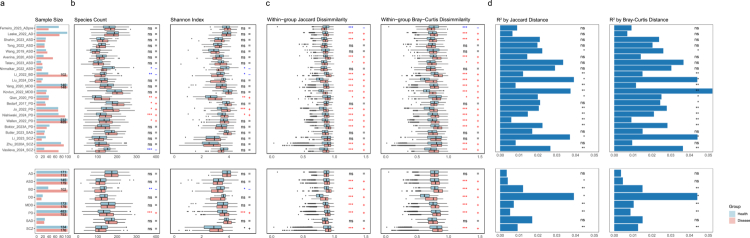
Characterization of gut microbial signatures in neuropsychiatric cohorts. a) Sample size per cohort and disease. b) Alpha diversity was quantified using species count and Shannon index measurements. c) Beta diversity differences measured through Jaccard and Bray-Curtis dissimilarity. d) Cohort-specific and disease-specific microbiome variation by calculating PERMANOVA R^2^ values through Jaccard and Bray-Curtis distance matrices. Figure elements utilize light blue bars or boxes for healthy control data, and pink bars or boxes for disease group data. The data in boxplots is represented using interquartile ranges (IQRs), with the median shown as a horizontal line, and the whiskers extending to the most extreme points within 1.5 times the IQR. Directional markers: “+” indicates disease-enriched species (red), “−” denotes health-enriched species (blue). Statistical significance: *p < 0.05, **p < 0.01, ***p < 0.001; ns (not significant, *p* > 0.05).

To systematically investigate the gut microbiota-disease interactions across neuropsychiatric disorders, we established a comprehensive analytical framework operating at both cohort and disease levels. Our analysis utilized a high-quality dataset (*n* = 2,734) generated through Illumina paired-end sequencing (Materials and methods, Figure 2a). To enhance analytical robustness, we used both relative abundance and prevalence metrics. Taxonomic profiling conducted using MetaPhlAn4 demonstrated consistent microbial community structures across all cohorts (Figure S2).

Ecological community structure was quantified via *α*-diversity indices and *β*-diversity dissimilarity matrices. Across cohorts, PD patients exhibited a significant elevation or upward trend in *α*-diversity compared to healthy controls, whereas other neuropsychiatric disorders demonstrated inconsistent or non-significant alterations. Integration of multiple PD cohorts revealed a significant increase in microbial alpha diversity (*p* = 2.47 × 10^−6^) (Figure 2b, Table S2). These findings challenge the “dysbiosis-as-depletion” paradigm.^58^ Furthermore, consistent *β*-diversity perturbations emerged in disease groups, with statistically significant increases in Jaccard and Bray-Curtis dissimilarities observed in 15 out of 22 cohorts (68% of studied populations) (Figure 2c, Table S2). Such patterns corroborate prior observations of disrupted microbial community architecture and reinforce the “dysbiosis” paradigm in neuropsychiatric disorders.^59^^,^^60^ PERMANOVA was performed with disease status as the explanatory factor to quantify its contribution (R²) to overall community variation. Five neuropsychiatric disorders - PD, SCZ, depressive disorder (DD), major depressive disorder (MDD), and bipolar disorder (BD) - demonstrated significant gut microbiome associations (PERMANOVA *p* < 0.05 for each disorder). Notably, autism spectrum disorder (ASD) showed significance only in the Wang_2019_ASD study, with observed significance specifically when using Jaccard distance-based R² calculations. Alzheimer’s disease (AD, including prodromal stages) and social anxiety disorder (SAD) had no significant microbiota-associated R^2^ relationships across cohorts (Figure 2d, Table S2).

### Differential species and pathways in neuropsychiatric disorders

To elucidate shared microbial signatures across neuropsychiatric disorders, we identified taxonomic and functional variations across cohorts and diseases using MaAsLin3^57^ on paired abundance and prevalence profiles. For disorder-specific differential analysis, we systematically evaluated three batch correction methods for taxonomic relative abundance data: ComBat, DEBIAS-M, and MMUPHin (Figure S3, 4) and PERMANOVA was performed with batch effect as the explanatory factor to quantify its contribution (R²) to overall community variation. Based on this comparative assessment, we ultimately implemented both DEBIAS-M and MMUPHin for integrated multi-cohort analysis following batch effect correction. These methods demonstrated effective batch effect reduction of 74.4% and 57.5%, respectively.

**Figure 3.**
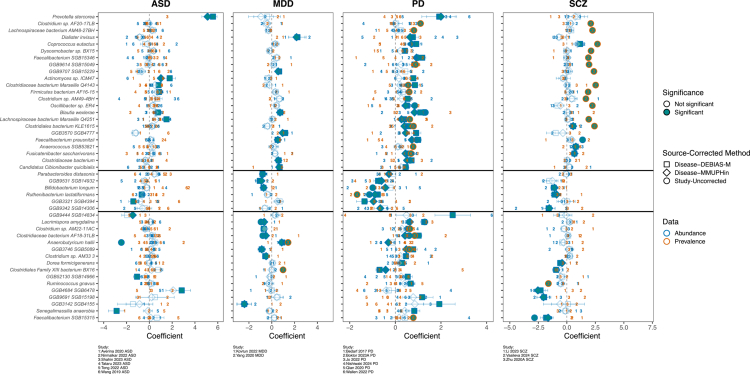
Microbial species alterations in individual and integrated cohorts by disease. Identification of differential species across individual cohorts and integrated disease cohorts. Numerical labels correspond to source studies. Geometric representations: Squares = DEBIAS-M-corrected aggregated cohorts per disease; Diamonds = MMUPHin-corrected aggregated cohorts; Circles = uncorrected single-study results. Border coding: Blue = abundance-based results; Orange = prevalence-based results. Significance threshold: Blue fill indicates FDR-adjusted *p*-value < 0.1. Stratification: Species above the first horizontal line are consistently enriched in controls across diseases (when significant); between lines indicates disease-enriched species; below the second line shows discordant directional changes.

Integrating these analytical approaches, we identified significant microbial alterations in four distinct neuropsychiatric disorders: ASD, MDD, PD and SCZ. Meta-analysis revealed 294 taxon-specific recurrence events with disease-associated enrichment patterns (FDR-adjusted *p* < 0.1), representing cumulative microbial associations with disease status across participating cohorts (Table S3). Within this set, 23 species exhibited health-associated enrichment across various disease conditions, while 6 species showed enrichment in disease cohorts, and 16 context-dependent species exhibited disorder-specific enrichment patterns (Figure 3, Table S3). Notably, PD and SCZ demonstrated the highest concordance in microbial alterations, sharing 15 health-enriched and 4 disease-enriched species (Figure S5). Most control-enriched species were short-chain fatty acid (SCFA)-producing species. For example, *Faecalibacterium prausnitzii*, a SCFA-producing bacterium,^61^ exhibited significant enrichment in healthy individuals across three independent diseases encompassing MDD, PD and SCZ patients. In contrast, *Ruthenibacterium lactatiformans* demonstrated consistent enrichment in patient cohorts across three independent diseases (ASD, PD and SCZ). These cross-disease conservation patterns highlight core microbial regulators of brain health, suggesting broad-spectrum therapeutic targets for restoring gut-brain axis homeostasis. We also found context-dependent microbial patterns, particularly for *Lacrimispora amygdalina* and the unclassified GGB3746_SGB5089, which exhibited diametrically opposite enrichment profiles across disorders. These species were significantly depleted in healthy controls relative to MDD patients, yet showed the reverse pattern in PDs. This bidirectional dysregulation suggests these microbes may play disease-specific roles - potentially as protective commensals in PD that become pathogenic in MDD, or alternatively, may reflect disorder-specific alterations in gut microenvironment favoring their proliferation.

Our meta-analysis identified significant alterations in 108 metabolic pathways across four neuropsychiatric disorders (ASD, MDD, PD, SCZ; FDR-adjusted *p* < 0.1), including 20 consistently health-enriched pathways, 27 disease-enriched pathways, and 16 context-dependent pathways showing disorder-specific patterns (Figure S6, Table S4). PD and SCZ exhibited the strongest pathway overlap, sharing 29 disease-enriched and 18 health-enriched pathways (Figure S5). Key findings included depletion of adenosylcobalamin salvage (COBALSYN.PWY) in MDD/PD and enrichment of GABA shunt (GLUDEG.I.PWY) in ASD/PD patients.

### Gut-brain diagnostic models for single diseases

**Figure 4.**
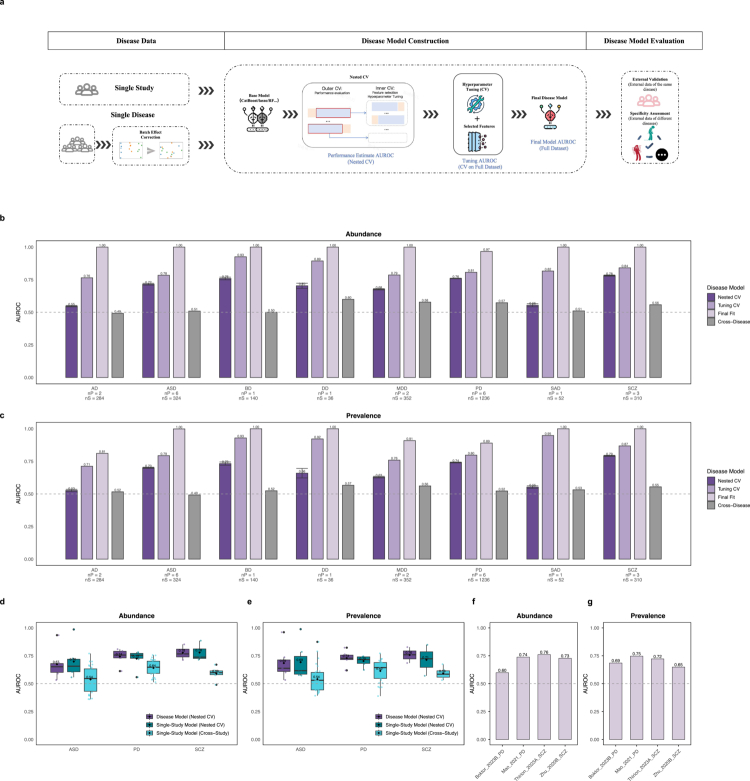
Disease-specific classifier construction and performance. a) The workflow begins with data preparation (multi-study integration with batch correction or direct use of single studies). A 5-fold nested cross-validation is employed for model optimization, performing feature selection and hyperparameter tuning in the inner loop. A final model is then trained on all data using the optimal parameters and subsequently evaluated on an external dataset. b−c) Bars show the disease classification performance (AUROC) of models using abundance (b) and prevalence (c) data. The deep purple is the mean unbiased Nested CV AUROC, estimating the performance of the entire model selection pipeline. And the error bars depict the standard deviation of these 10 repeats. The purple is the Tuning CV AUROC, derived from a standard 5-fold CV using the final feature selected via nested CV. The light purple shows performance on full data set. Gray bars represent the mean AUROC on non-target diseases, indicating cross-disease specificity. d) Boxplots show the distribution of model performance (AUROC) across individual studies for a given disease. Each point within a plot represents the result for a single study. Purple shows the performance of the integrated single-disease model on a per-study basis, where the AUROC for each study is calculated from the outer loop predictions obtained during the model's Nested CV. Green: The unbiased Nested CV AUROC for each single-study model, calculated using only its own respective dataset. Cyan: Cross-study generalization, representing the AUCs when each single-study model is used to predict all other studies of the same disease.

To address the challenge of inconsistent diagnostic accuracy and poor generalizability of microbiome-based diagnostic models, we developed a standardized framework termed MetaClassifier to optimize model construction and evaluation (see Materials and Methods). For our disease-specific analyzes, we employed the comprehensive workflow illustrated in Figure 4a. This workflow begins with an initial data harmonization step, involving the integration and batch correction of multi-study data where applicable. Following this, the core modeling and validation stages are executed using the MetaClassifier framework, which encompasses nested CV for feature selection and the final external validation of the model's generalization and specificity (see Materials and Methods).

A comparison of five models across individual studies indicated that abundance-based CatBoost achieved the best overall predictive performance mean nested CV AUROC = 0.71 (Figure S7a,b). However, these single-study models exhibited limited generalizability and dropped to near-random levels when applied to independent cohorts of the same disease, a finding that held for models based on both abundance and prevalence data (Figure S7c-f, Table S5). To overcome this, we integrated studies of same disease and applied batch correction, subsequently identifying a DEBIAS-M-corrected CatBoost model as optimal for abundance data and a CatBoost model for prevalence data through rigorous re-benchmarking (Figure S8, Table S6).

Our framework successfully generated robust predictive models for a majority of the diseases studied. For abundance data, six of the eight diseases achieved nested cross-validation AUROCs at or exceeding 0.7. Across all eight diseases, the tuning cross-validation AUROCs were consistently high at approximately 0.8, and the final fit AUROCs approached 1.0, reflecting the model's fit to the full high-quality dataset (Figure 4b). Therefore, to assess robust generalization performance, we prioritized the nested cross-validation results within the high-quality dataset (Figure 4b) and independent external validation using the platform-extended dataset (Figure 4f,g). Prevalence-based models demonstrated a comparable level of performance (Figure 4c). Critically, all predictive models proved to be highly specific, showing only random-chance performance when applied to other diseases (mean cross-disease AUC ≈ 0.5) (Figure 4b,c).

The integration of disease-specific studies was crucial for improving model generalizability over single-project approaches. Our single-study model analysis of diseases ASD, PD, and SCZ, each with at least three cohorts, quantitatively demonstrated this necessity (Figure 4d–e). While models trained on a single study performed poorly when tested across studies (mean AUC dropping from 0.74 to 0.59), our integrated models maintained consistently high and robust performance across all individual studies (mean AUROC = 0.73). This proves that our integration strategy is essential to overcome cross-study heterogeneity within the same disease. Notably, the disease-specific classifiers also demonstrated considerable transferability to a platform-expended dataset comprising independent external cohorts, achieving AUROCs of approximately 0.70 for PD and SCZ (Figure 4f,g). Besides, shared microbial features emerged across diagnostic classifiers. For instance, *Bifidobacterium longum* enrichment emerged as a discriminative feature in SCZ discrimination models and was also identified as a key differentiator in abundance-based classifiers for AD, ASD, MDD, PD, and SCZ, as well as prevalence-based classifiers for ASD, AD, PD and SCZ (Figure S9, 10).

### The Microbial Gut-Brain Axis Health Index

To inform preventive interventions for neuropsychiatric disorders and promote long-term brain health, early detection and risk stratification of neuropsychiatric status are essential. We developed the Microbial Gut-Brain Axis Health Index (MGBA-HI), a data-driven indicator to provide a single, quantitative score reflecting the overall microbial signature that distinguishes the neuropsychiatric and disease state from controls (Materials and methods).

**Figure 5.**
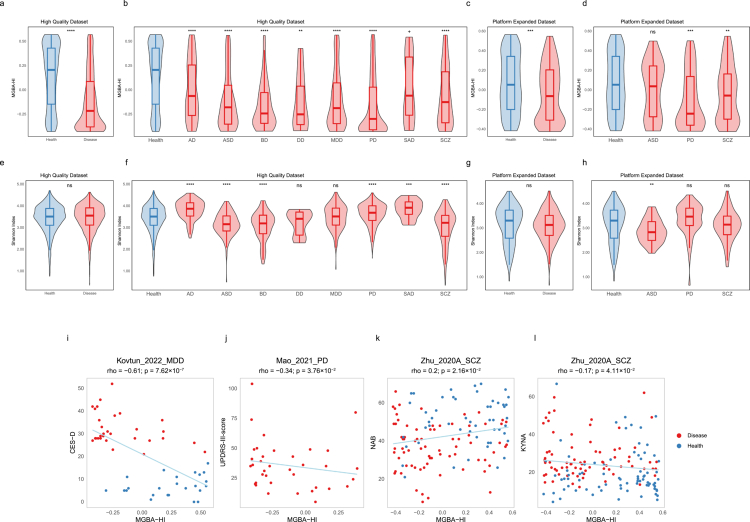
MGBA-HI evaluation in neuropsychiatric disorders. a−b) Comparative distributions of MGBA-HI and Shannon index between healthy (blue) and disease (pink) groups in both High-Quality (*n* = 2,734) and Platform expanded (*n* = 400) datasets. Statistical significance assessed by Wilcoxon rank-sum test. ****p ≤ 0.0001; ***p ≤ 0.001; **p ≤ 0.01; *p ≤ 0.05; + 0.05 < *p* < 0.1; ns *p* ≥ 0.1. c) Clinical correlation analysis showing MGBA-HI association with diagnostic phenotypes. Spearman's correlation coefficients (rho) and corresponding *P*-values are displayed. Locally weighted smoothing lines illustrate trend relationships.

The MGBA-HI demonstrated exceptional discriminative power in the high-quality dataset, with patients scoring significantly lower than healthy controls (Wilcoxon rank-sum *p* < 2.22 × 10^−16^, Cliff’s delta = –0.45), achieving significant separation for every individual disorder (Figure 5a,b, Table S7). This diagnostic capability extended to the more heterogeneous, platform-extended dataset (*p* = 4.25 × 10^−4^, Cliff’s delta = –0.21), where it maintained significance for PD (*p* = 2.06 × 10^−4^, Cliff’s delta = –0.34) and SCZ (*p* = 3.65 × 10^−3^, Cliff’s delta = –0.20) (Figure 5c,d). Remarkably, the MGBA-HI also showed powerful generalizability to disorders entirely excluded from model training, successfully differentiating patients with anorexia nervosa (AN; *p* = 8.12 × 10^−4^, Cliff’s delta = –0.28), obsessive-compulsive disorder (OCD; *p* = 3.19 × 10^−3^, Cliff’s delta = –0.35) in a disease-extended dataset (see Materials and Methods, Figure S11). In stark contrast, the conventional Shannon alpha-diversity index failed to distinguish patients from controls across these same scenarios. It showed no significant differences between the overall patient cohort and healthy controls, nor within the DD and MDD subgroups (Figure 5e,f). This limitation persisted in the platform-extended dataset for PD and SCZ (Figure 5g,h) and for all tested disorders in the disease-extended dataset (Figure S11). These consistent, opposing outcomes underscore a key advantage of the MGBA-HI: its superior sensitivity in detecting disease-associated microbial signatures that are missed by traditional diversity measures.

Beyond its diagnostic accuracy, MGBA-HI scores showed robust correlations with key clinical biomarkers across multiple conditions. In MDD, MGBA-HI scores exhibited a strong inverse correlation with depression severity (Kovtun_2022_MDD, CES-D: rho = –0.61, *p* = 7.62 × 10^−7^) and motor deficits in PD (Mao_2021_PD, UPDRS-III: rho = –0.34, *p* = 3.76 × 10^−2^). Furthermore, in SCZ (Zhu_2020A_SCZ), higher MGBA-HI scores were associated with better overall neurocognitive performance, as measured by the NAB (rho = 0.20, *p* = 2.16 × 10^−2^), and lower KYNA (rho = –0.17, *p* = 4.11 × 10^−2^), suggesting a potential association with neurotransmitter regulation, a hypothesis that warrants further mechanistic investigation (Figure 5i-l, Table S8).

### Core neuropsycho-protective microbiota

**Figure 6.**
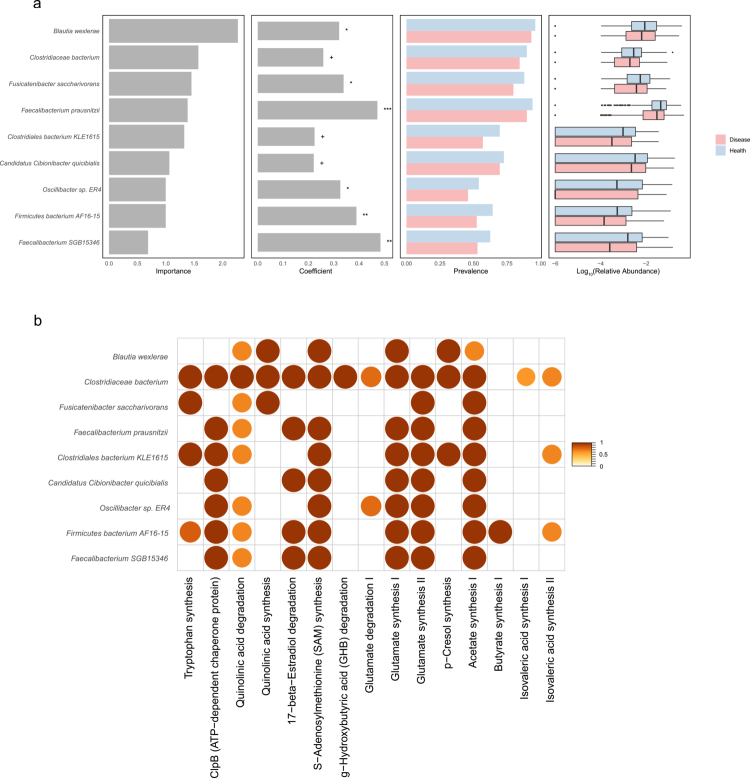
The characteristics of core neuropsycho-protective microbiota. a) Multi-parameter visualization of 9 core neuropsycho-protective species showing: MGBA-HI feature importance scores, coefficient, prevalence rates across cohorts and relative abundance distributions. healthy controls = light blue, disease = pink). ***p ≤ 0.001; **p ≤ 0.01; *p ≤ 0.05; + 0.05 < *p* < 0.1. b) Gut-brain module annotations for species-level genome bins corresponding to 9 high-priority targets from MetaPhlAn4, filtered by > 0.67 module coverage threshold.

The cross-disease conservation patterns highlighted core microbial species as regulators of brain health, critical for maintaining neurological health. We integrated health-enriched or high-prevalence species in healthy individuals compared with patients of neuropsychiatric disorders, and MGBA-HI-related species, to systematically delineate the core microbiota signature as the protective indicator of neuropsychiatric health. Differential abundance analysis identified 23 health-associated species (Figure 3). The MGBA-HI subsequently prioritized 107 species with robust discriminative capacity for healthy individuals, while prevalence screening revealed 73 microbial features consistently colonizing ≥50% of healthy individuals (Table S9). The intersection of these species ultimately defined 9 core neuropsycho-protective species (Figure 6a, Table S9).

To explore the neuroactive potential of the core protective species, we conducted genome-scale metabolic reconstruction of 21 genomes within the core consortium. Functional interrogation revealed that most genomes harbor complete gut–brain modules (GBMs)^56^ including SCFAs synthesis, amino acid metabolism, and the synthesis of several neurotransmitters. Notably, extensive functional overlap has been revealed among the 9-core gut bacterial species. All 9 species maintained genetic capacity for both glutamate and acetate biosynthesis, while 8 species (88.9%) encoded S-adenosylmethionine (SAM) synthesis pathways. *Firmicutes bacterium AF16-15* exhibits genetic potential for butyrate synthesis among the core species (Figure 6b, Table S10).

## Discussion

Gut-brain axis dysregulation is a hallmark of neuropsychiatric disorders, often accompanied by perturbations in gut microbial communities. However, current investigations into the role of the gut microbiome in these diseases face significant methodological constraints. To address these challenges, we established the GutMIND database, which integrates standardized preprocessing pipelines, systematic batch effect correction, and multivariate covariate calibration (e.g., age, sex) to achieve high-resolution characterization of cross-cohort microbial architectures. Our analytical framework synergizes community ecological dynamics with both taxon and functional-level discriminative features, enabling systematic elucidation of disease-specific microbial signatures. The diagnostic model employs a customizable architecture to optimize algorithm selection across diverse clinical datasets. Beyond mere disease classification, a significant finding of this study is the ability of MGBA-HI to capture differences between disease cohorts that were missed by conventional alpha-diversity metrics. This highlights the value of such targeted, data-driven approaches for revealing clinically meaningful microbial patterns in gut-brain axis disorders and offering a novel paradigm for precision medicine, public health strategies. Notably, the 9-core neuro-protective species identified herein exhibit dual biological plausibility: their ubiquitous presence in healthy guts underscores foundational barrier functions,^61^ while their convergent depletion across neuropsychiatric disorders hints at regulatory roles for microbial-derived metabolites (e.g., acetate, glutamate) in neuromodulatory pathways. It’s functional redundancy in neurotransmitter and SCFAs synthesis suggests a metabolic buffering capacity against perturbations in neuropsychiatric disorders, positioning the core consortium as a potential metabolic hub in neurophysiological regulation. These findings suggest that these species may serve as candidate targets for future “psychobiotic” development and therapeutic strategies.

The interpretation of this meta-analysis is framed by the heterogeneity inherent in its aggregated public datasets. A core limitation is the suboptimal sample size for certain disease cohorts, which may lead to variations in statistical power across conditions, and the lack of standardized reporting for key confounders, such as host ethnicity, diet, and medication, across studies. Baseline microbial signatures are known to vary with host ethnicity, and their association with clinical symptoms may not be uniform across populations.^62^ This is further compounded by the powerful influence of diet, a factor rarely standardized in the included studies.^63^^,^^64^ Critically, the widespread use of psychotropic medication, which can directly alter the microbiome and its functions, makes it difficult to disentangle the effects of treatment from the underlying disease pathology.^65^ This incomplete metadata creates a significant risk of residual confounding and hinders a comprehensive dissection of confounder-microbiome-disease associations. Besides, inconsistent diagnostic criteria for neuropsychiatric disorders across studies obscure potentially convergent biological signals. To disentangle the specific signal of disease from these effects, future prospective studies must incorporate standardized data collection frameworks, consistent diagnostic criteria, and longitudinal sampling to track changes over time. Moreover, such studies should prioritize the recruitment of drug-naïve patients to eliminate medication as a major confounder.

Our analytical decisions, while ensuring robustness, also define the boundaries of our conclusions. We excluded individuals with a BMI > 30 kg/m² to control for the powerful effects of obesity. Consequently, our findings may have limited applicability to this comorbid patient subgroup. Similarly, filtering out low-abundance species and studies with fewer than 10 samples, while necessary for statistical stability, means the potential role of rare microbes remains an open question. Furthermore, our analytical scope was confined to bacterial species, overlooking critical interkingdom interactions (e.g., with fungi and viruses) that could be pivotal to the gut-brain axis.

Finally, the study’s design imposes broader limitations. First, metagenomic data identifies genetic potential, not functional activity, meaning the actual expression of the neuroactive pathways we identified remains unconfirmed. This is intrinsically linked to the study's most fundamental limitation: its correlational nature. The reliance on cross-sectional data makes it impossible to distinguish cause from consequence, and the host side of the gut-brain axis, key processes like metabolite absorption and blood-brain barrier transport, remain unobserved. Establishing causality will therefore require an integrative research strategy that combines metagenomics with functional readouts like metatranscriptomics and metabolomics (e.g., profiling short-chain fatty acids and neurotransmitters). Such multi-omics approaches, when coupled with longitudinal designs and mechanistic interrogation through interventions like fecal microbiota transplantation or psychobiotics, will be vital for unlocking the therapeutic potential of the gut-brain axis.

In summary, our study constitutes a paradigm-shifting expansion of the microbiota-gut-brain axis theoretical framework through the GutMIND database, which for the first time integrates harmonized multi-cohort shotgun metagenomic data across eight neuropsychiatric disorders. By establishing the MetaClassifier diagnostic workflow and MGBA-HI health index, we validate the predictive utility of gut microbial signatures while identifying core protective species with convergent metabolic pathways. Overall, the GutMIND database advances traditional research on the microbiota-gut-brain axis by developing a transdiagnostic microbial framework, setting a methodological benchmark for cross-cohort comparability, and offering a mechanistic foundation for the translation of microbiome-based diagnostics and therapeutics in the context of neuropsychiatric disorders. Future investigations, including prospective clinical validation and mechanistic experiments, are now warranted to translate these microbiome-based insights into precision interventions.

## Supplementary Material

Table.zip Table.zip

Supplementary.pdf KGMI_A_2630563_SM5761.pdf

## Data Availability

MetaClassifier is publicly available at https://github.com/juyanmei/MetaClassifier. These related public data were derived from the following resources available in the public domain: NCBI (https://www.ncbi.nlm.nih.gov/sra) and CNSA (https://db.cngb.org/cnsa/). The related tables are available at https://figshare.com/s/416eae4ed99b8787304b.
