# Supplementary material for: GutMIND: A multi-cohort machine learning framework for integrative characteristics of the microbiota-gut-brain axis in neuropsychiatric disorders
Source: Gut Microbes. 2026 Feb 16;18(1):2630563. doi: 10.1080/19490976.2026.2630563 (PMC12915850; doi:10.1080/19490976.2026.2630563)
Supplement: Supplementary.pdf — KGMI_A_2630563_SM5761.pdf [file KGMI_A_2630563_SM5761.pdf]

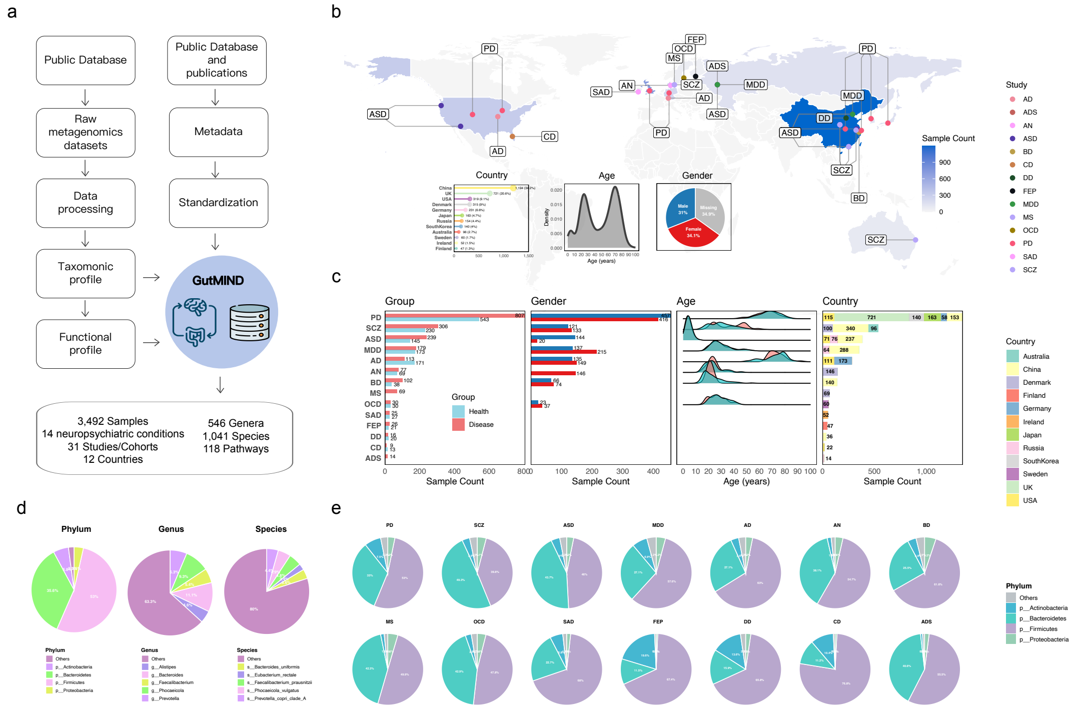

Figure 1. Overview of the GutMIND database

a) Workflow for the construction of the GutMIND database and core characteristics.

b) Demographic characteristics of GutMIND including geographic distribution, age stratification, and gender ratio.

c) Disease-specific features showing sample size, gender distribution, age range, and geographical coverage for each diagnostic category.

d) Microbial composition of the GutMIND cohort across taxonomic levels (Phylum, Genus, Species).

e) Phylum-level microbial signatures associated with different disease states.

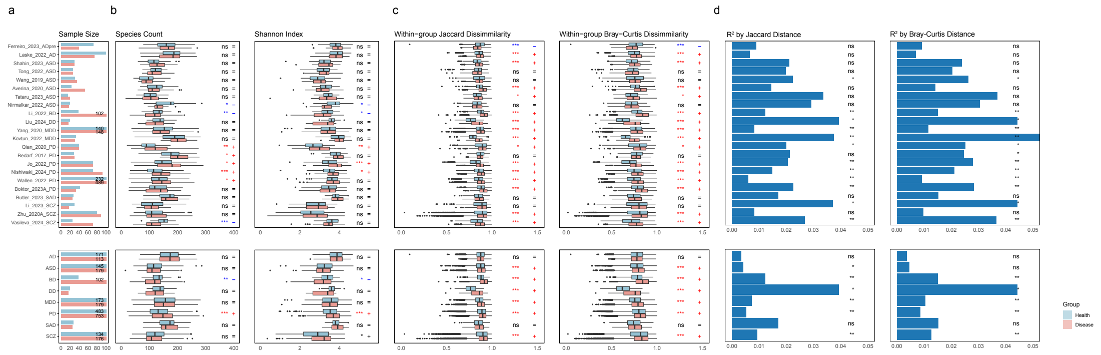

Figure 2. Characterization of gut microbial signatures in neuropsychiatric cohorts

a) Sample count for single cohort and single disease

b) Alpha diversity was quantified using species count and Shannon index measurements.

c) Beta diversity differences measured through Jaccard and Bray-Curtis dissimilarity.

d) Cohort-specific and disease-specific microbiome variation by calculating PERMANOVA R2 values through Jaccard and Bray-Curtis distance matrices.

Figure elements utilize light blue bars or boxes for healthy control data, and pink bars or boxes for disease group data. The data in boxplots is represented using interquartile ranges (IQRs), with the median shown as a horizontal line, and the whiskers extending to the most extreme points within 1.5 times the IQR. Directional markers: "+" indicates disease-enriched taxa (red), "-" denotes health-enriched taxa (blue). Statistical significance: P < 0.05, *P < 0.01, **P < 0.001; ns (not significant, P > 0.05).

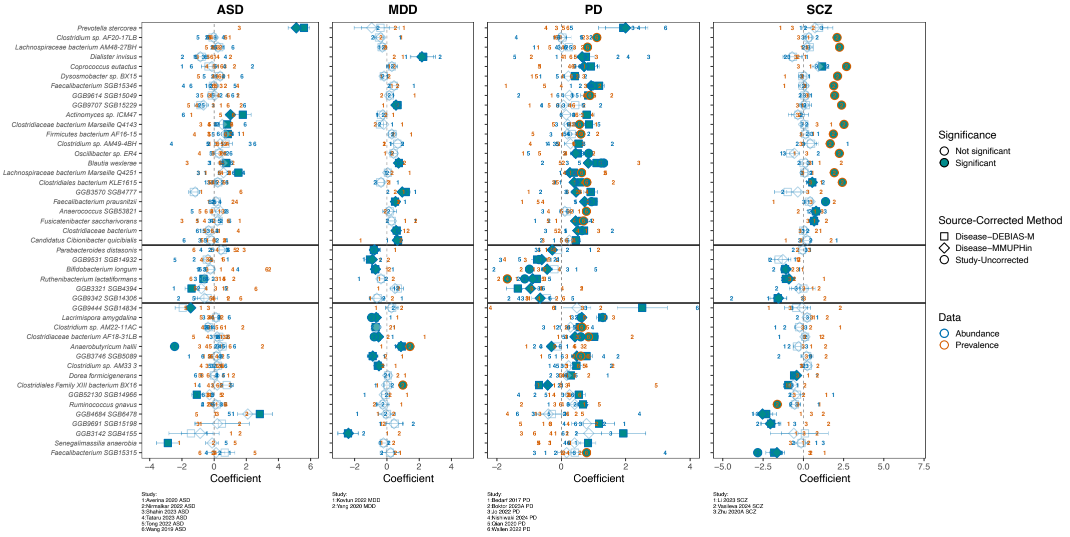

Figure 3. Microbial species alterations in single cohort and integrated cohorts within disease

Identification of differentially species across individual cohorts and integrated disease Cohorts. Numerical labels correspond to source studies. Geometric representations: Squares = DEBIAS-M-corrected aggregated cohorts per disease; Diamonds = MMUPHin-corrected aggregated cohorts; Circles = uncorrected single-study results. Border coding: Blue = abundance-based results; Orange = prevalence-based results. Significance threshold: Blue fill indicates FDR-adjusted P-value < 0.1. Stratification: Species above the first horizontal line are consistently enriched in controls across diseases (when significant); between lines indicates disease-enriched species; below the second line shows discordant directional changes.

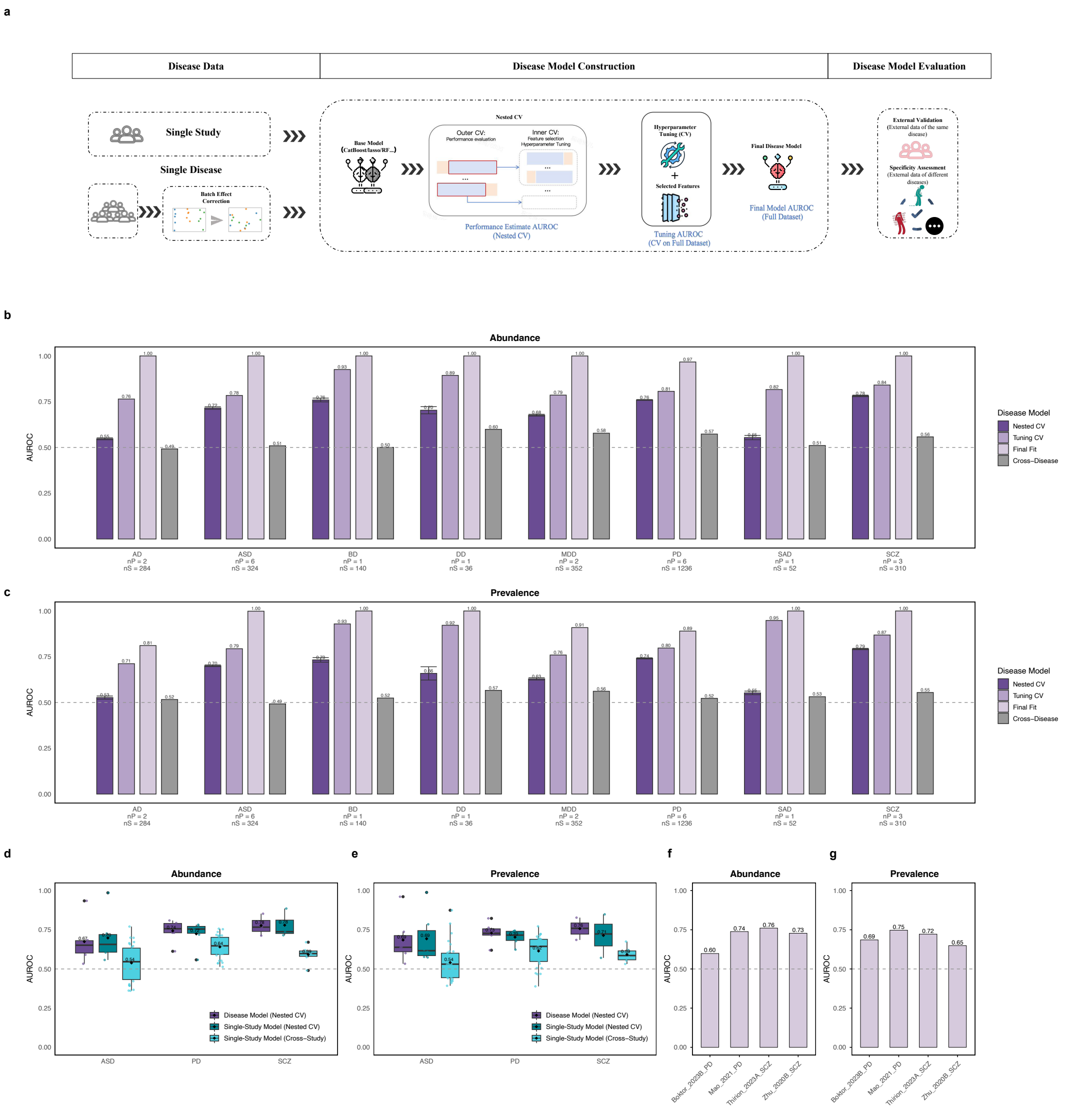

Figure 4. Disease-specific classifier construction and performance

a) The workflow begins with data preparation (multi-study integration with batch correction or direct use of single studies). A 5-fold nested cross-validation is employed for model optimization, performing feature selection and hyperparameter tuning in the inner loop. A final model is then trained on all data using the optimal parameters and subsequently evaluated on an external dataset.

b-c) Bars show the disease classification performance (AUROC) of models using abundance (b) and prevalence (c) data. The deep purple is the mean unbiased Nested CV AUROC, estimating the performance of the entire model selection pipeline. And the error bars depict the standard deviation of these 10 repeats. The purple is the Tuning CV AUROC, derived from a standard 5-fold CV using the final feature selected via nested CV. The light purple shows performance on full data set. Gray bars represent the mean AUROC on non-target diseases, indicating cross-disease specificity.

d) Boxplots show the distribution of model performance (AUROC) across individual studies for a given disease. Each point within a plot represents the result for a single study. Purple shows the performance of the integrated single-disease model on a per-study basis, where the AUROC for

each study is calculated from the outer loop predictions obtained during the model's Nested CV. Green: The unbiased Nested CV AUROC for each single-study model, calculated using only its own respective dataset. Cyan: Cross-study generalization, representing the AUCs when each single-study model is used to predict all other studies of the same disease.

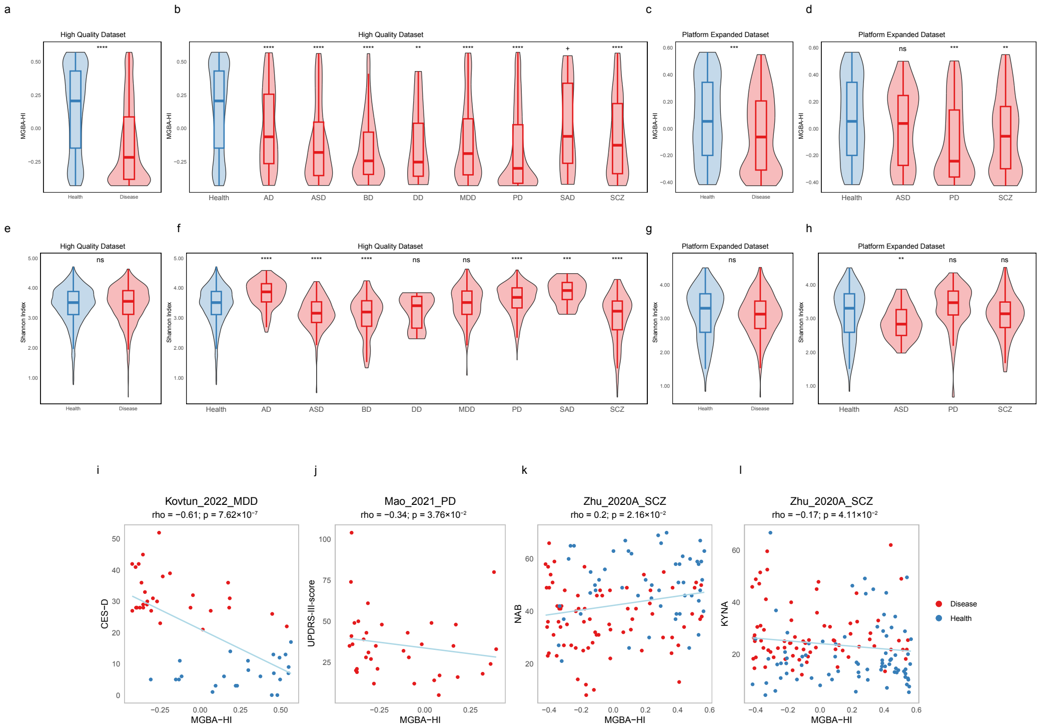

Figure 5. MGBA-HI evaluation in neuropsychiatric disorders

a-b) Comparative distributions of MGBA-HI and Shannon diversity indices between healthy (blue) and disease (pink) groups in both High-Quality (n=2,734) and Platform expended (n=400) datasets. Statistical significance assessed by Wilcoxon rank-sum test. **** $p \leqslant 0.0001$; *** $p \leqslant 0.001$; ** $p \leqslant 0.01$; * $p \leqslant 0.05$; + $0.05 < p < 0.1$; ns $p \geqslant 0.1$.

c) Clinical correlation analysis showing MGBA-HI association with diagnostic phenotypes. Spearman's correlation coefficients (rho) and corresponding P-values are displayed. Locally weighted smoothing lines illustrate trend relationships.

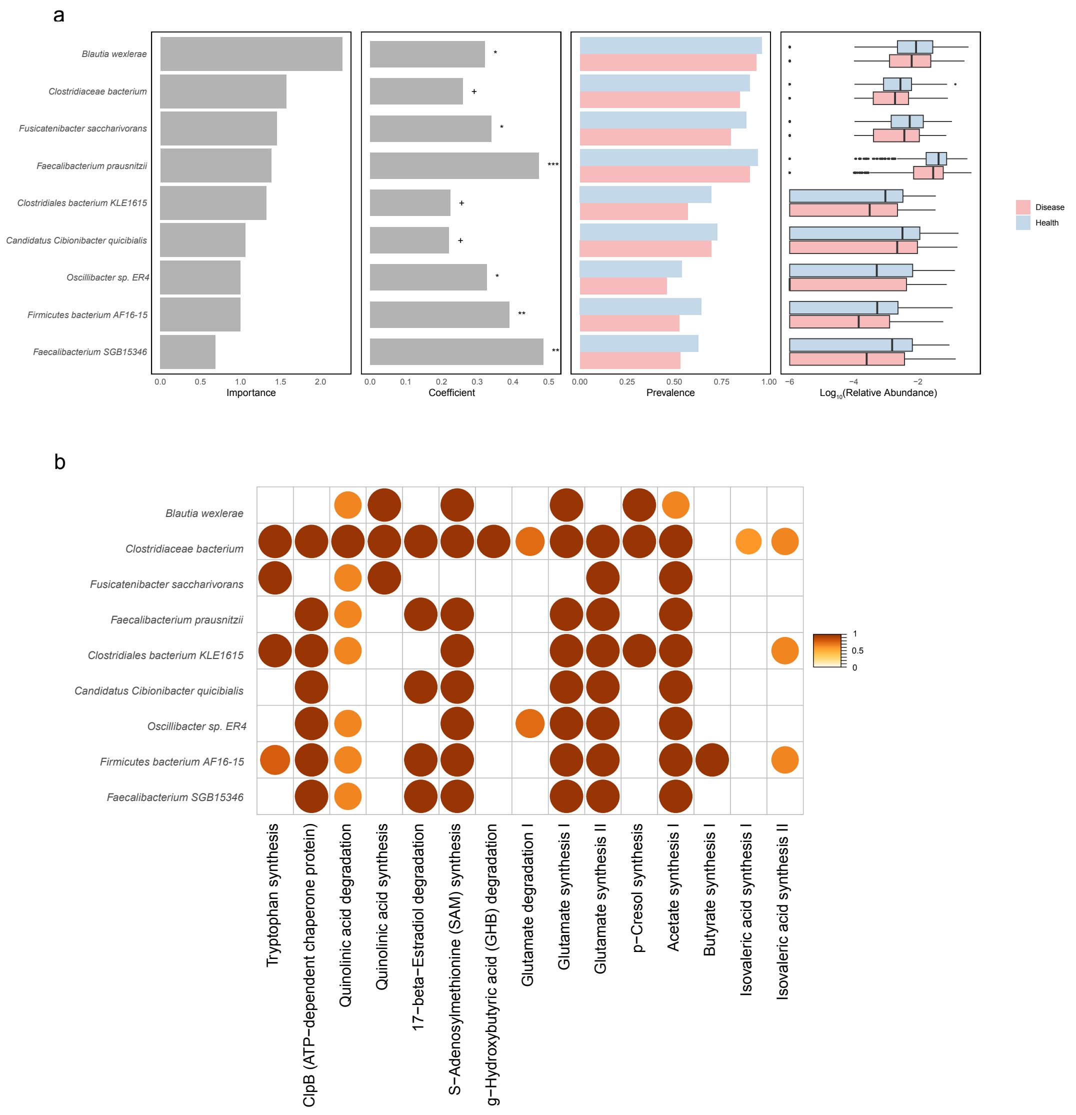

Figure 6. The characteristics of core neuropsycho-protective microbiota

a) Multi-parameter visualization of 9 core neuropsycho-protective species showing: MGBA-HI feature importance scores, coefficient, prevalence rates across cohorts and relative abundance distributions. healthy controls=light blue, disease=pink). ** p ⩽ 0.01; * p ⩽ 0.05; + 0.05 < p < 0.1.

b) Gut-brain module annotations for species-level genome bins corresponding to 9 high-priority targets from MetaPhlAn4, filtered by >0.67 module coverage threshold.

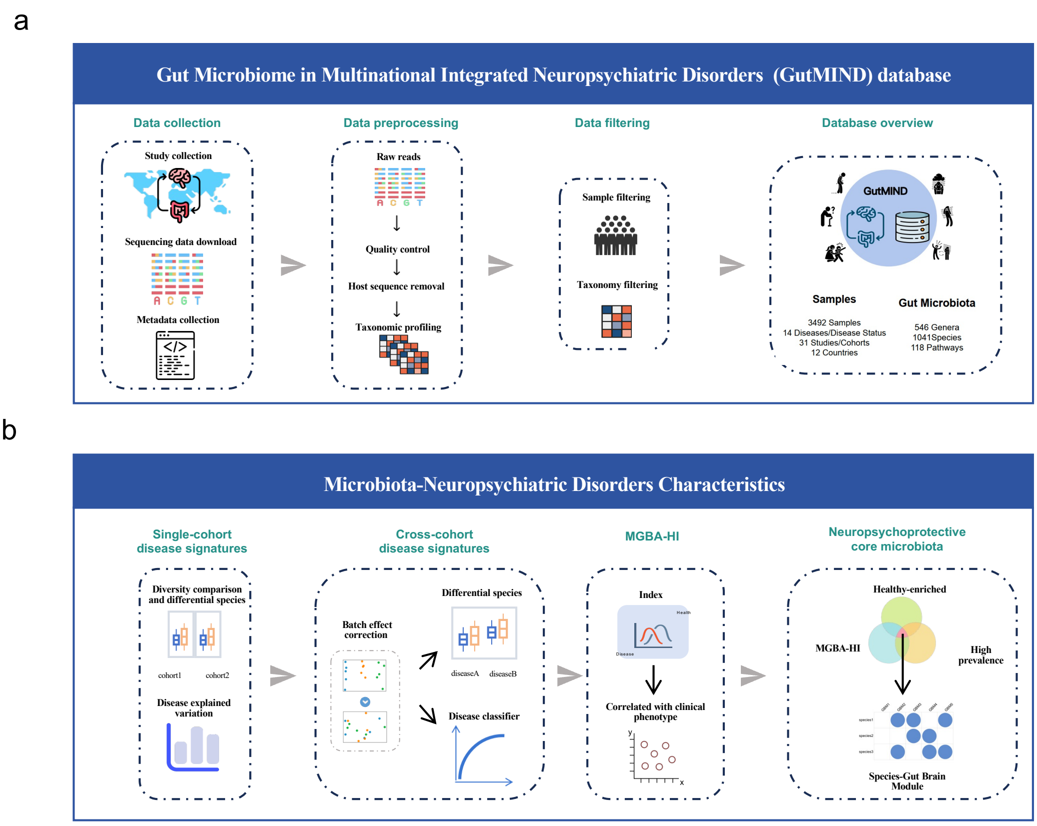

Figure S1. Workflow for fecal metagenomic GutMIND database construction and cross-cohort integrated analysis in neuropsychiatric disorders

a) We systematically collected 31 global metagenomic cohort studies encompassing neuropsychiatric disorders. Sequencing data underwent standardized bioinformatics processing, including quality control, host DNA depletion, and taxonomic annotation. Following the implementation of stringent inclusion criteria through a multi-step quality assurance framework, the final GutMIND database integrated 31 high-confidence studies spanning 12 countries, comprising 3,492 samples across 14 neuropsychiatric conditions.

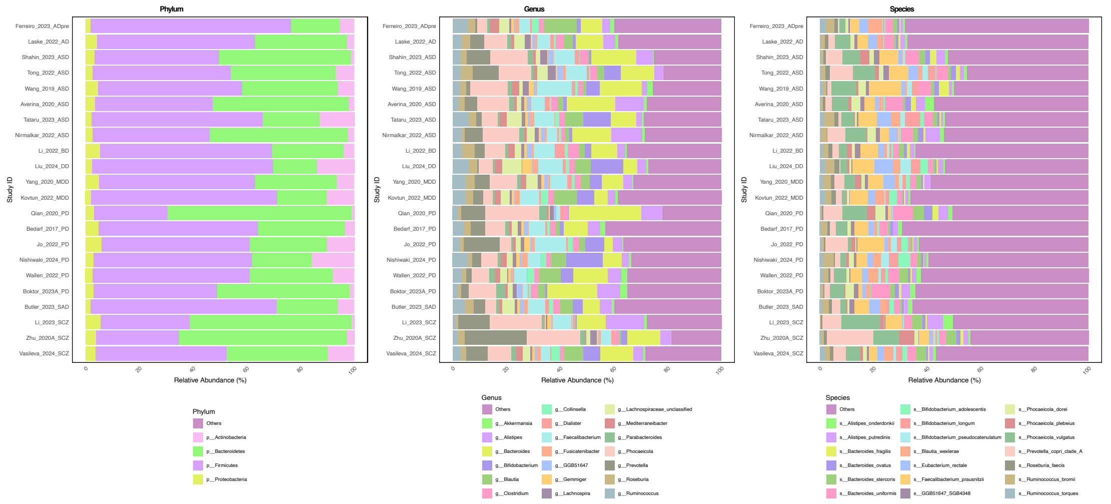

Figure S2. Taxonomic relative abundance profiles of high-quality cohort

Stacked bar plots demonstrating phylum-level (a), genus-level (b), and species-level (c) relative abundance profiles.

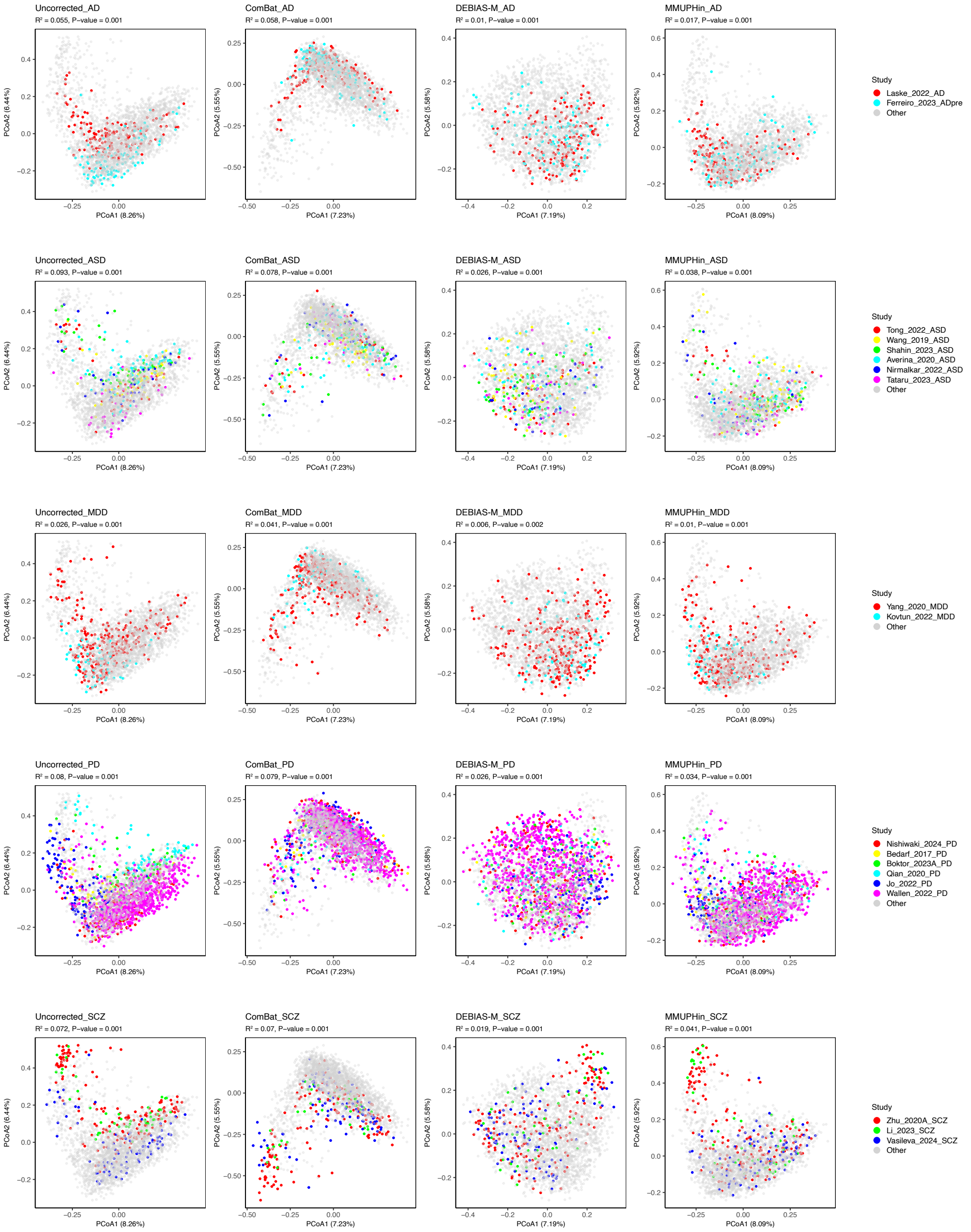

Figure S3. Microbial community variation before and after batch correction

Principal coordinates analysis (PCoA) plots and corresponding PERMANOVA R^2 values demonstrate the effects of different batch correction methods (uncorrected, ComBat, DEBIAS-M, MMUPHin) across distinct neuropsychiatric disorders. Each row represents a specific disease entity, enabling direct comparison of correction efficacy.

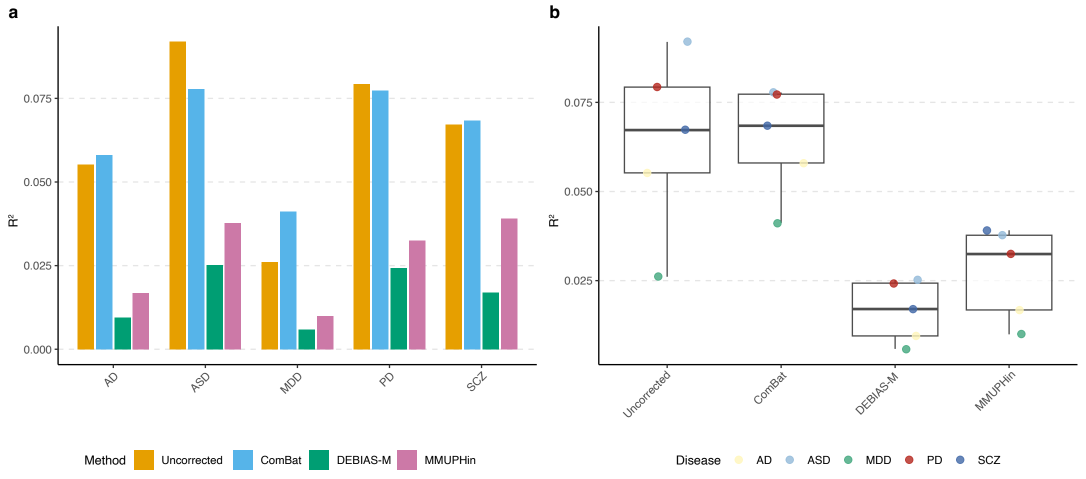

Figure S4. Quantitative assessment of batch effects before and after correction

a) Bar plot comparing batch-associated variation (PERMANOVA R^2) across different correction methods (uncorrected, ComBat, DEBIAS-M, MMUPHin) for each neuropsychiatric disorder.

b) Boxplot summarizing the distribution of R^2 values across all diseases, showing median (central line), interquartile range (boxes), and 1.5×IQR ranges (whiskers).

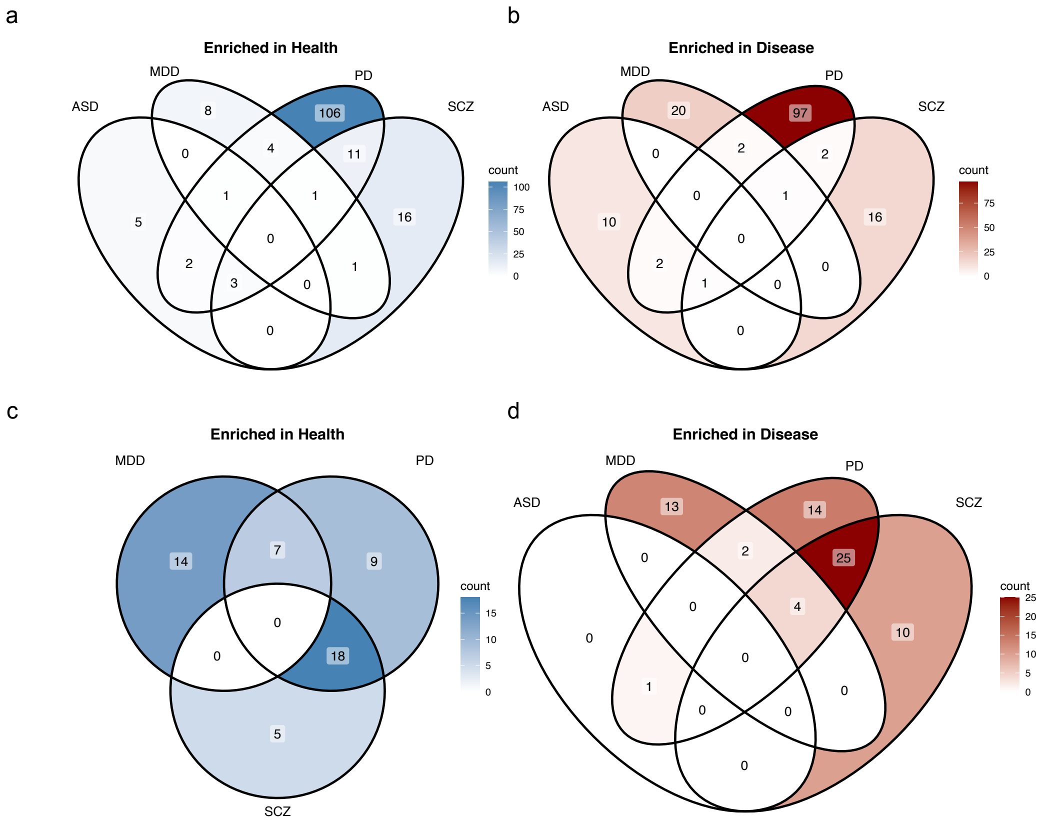

Figure S5. Shared and distinct microbial species and functional pathways across neuropsychiatric disorders

a-b) Venn diagrams showing overlaps of (a) health-enriched and (b) disease-enriched bacterial species across different disease.

c-d) Venn diagrams demonstrating intersections of (c) health-enriched and (d) disease-enriched metabolic pathways across different disease.

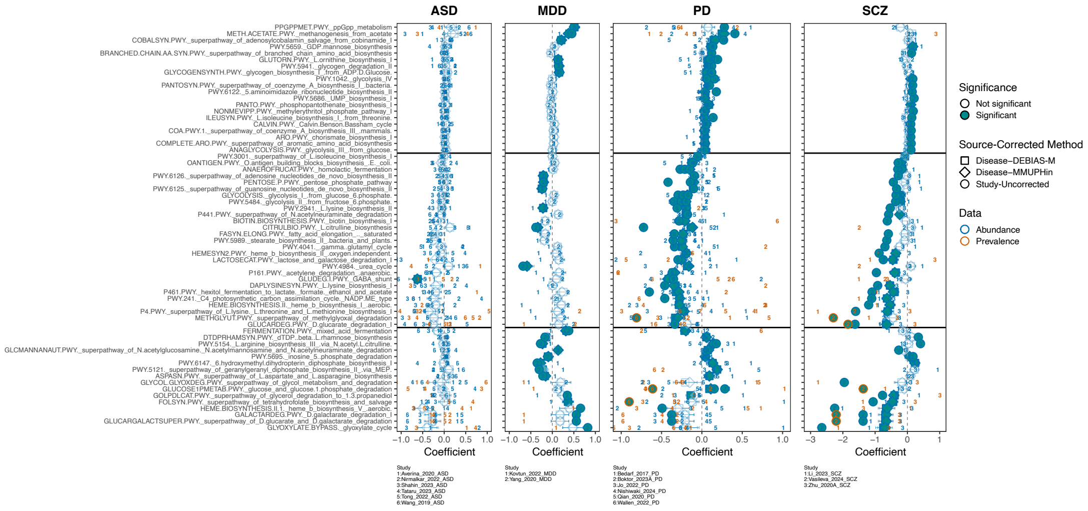

Figure S6. Functional pathway alterations in single and integrated disease cohorts

Identification of differentially pathways across individual cohorts and integrated disease Cohorts. Numerical labels correspond to source studies. Geometric representations: Squares = DEBIAS-M-corrected aggregated cohorts per disease; Diamonds = MMUPHin-corrected aggregated cohorts; Circles = uncorrected single-study results. Border coding: Blue = abundance-based results; Orange = prevalence-based results. Significance threshold: Blue fill indicates FDR-adjusted P-value < 0.1. Stratification: Species above the first horizontal line are consistently enriched in controls across diseases (when significant); between lines indicates disease-enriched species; below the second line shows discordant directional changes.

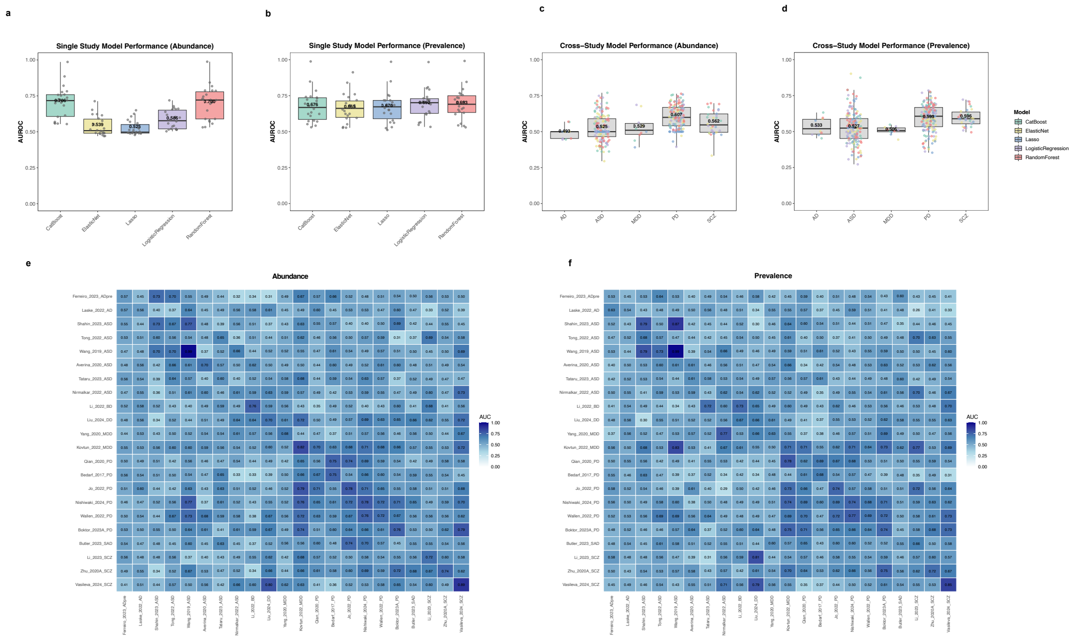

Figure S7. Disease classification performance of single-cohort models

a-b) Single study discriminative power (AUROC) of different machine learning methods using (a) species abundance data and (b) species prevalence data. The AUROC is the 5-fold nested cross-validation of the outer loop.

c-d) Cross-study validation performance (AUROC) for the same disease across different cohorts, evaluated with (c) abundance-based models and (d) prevalence-based models.

e-f) Model generalizability across all cohorts assessed by (e) abundance-based CatBoost models and (f) prevalence-based CatBoost models, presented as AUROC distributions. Each row represents a model trained exclusively on a single study, and each column represents the study used for testing. The AUROC values on the diagonal represent the unbiased performance estimate for each model within its own study, calculated via a 5-fold nested cross-validation.

.

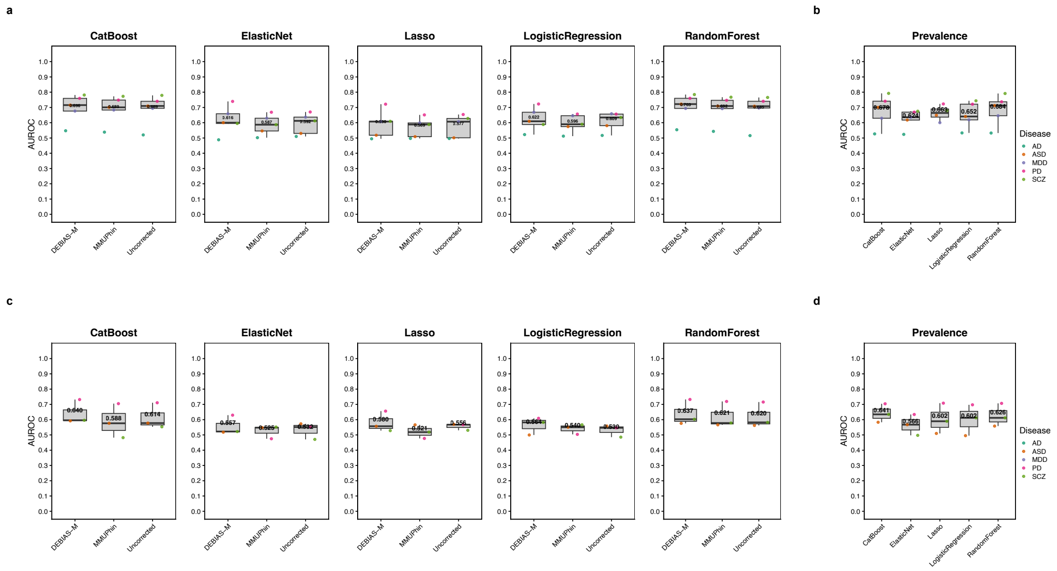

Figure S8. Performance evaluation of disease-specific classification models

a) Comparative 5-fold nested cross-validation AUROC values of different machine learning models before and after batch correction (multiple methods) using relative abundance profiles.

b) Model performance 5-fold nested cross-validation (AUROC) across machine learning algorithms using prevalence data with 5-fold nested cross-validation.

c) Leave-one-cohort-out (LOCO) results showing correction method effects on model performance using relative abundance features.

d) LOCO validation performance of various models using prevalence-based features.

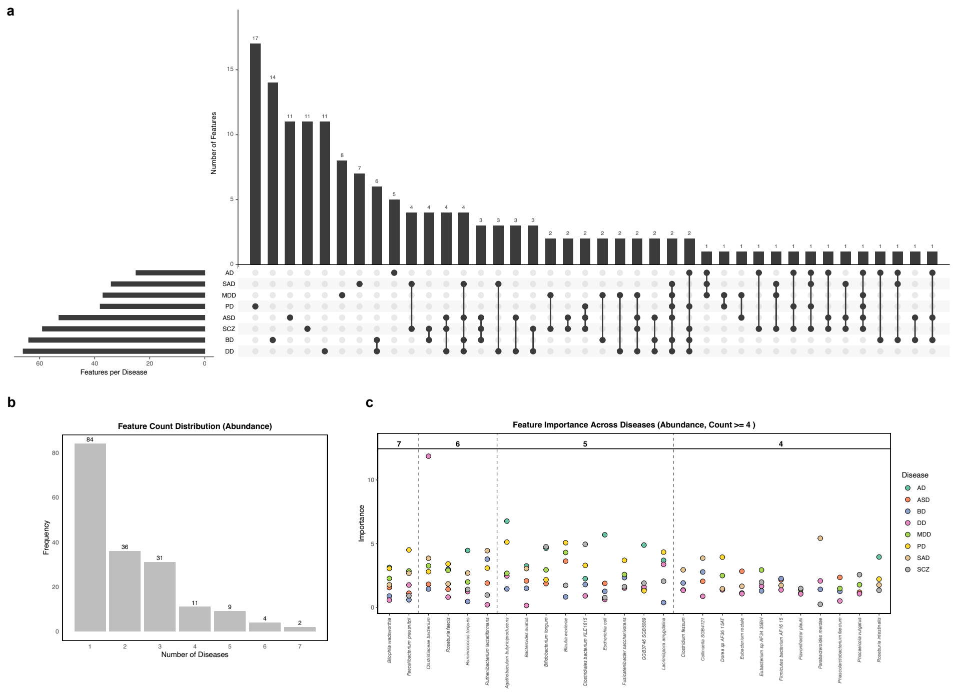

Figure S9. Selected features of disease-specific classifiers based on abundance data

a) Upset analysis depicting the intersections of features across different disease-specific diagnostic models.

b) Number of feature intersections within each disease-specific classifier.

c) Specific features identified in each disease-specific classifier (n > 4).

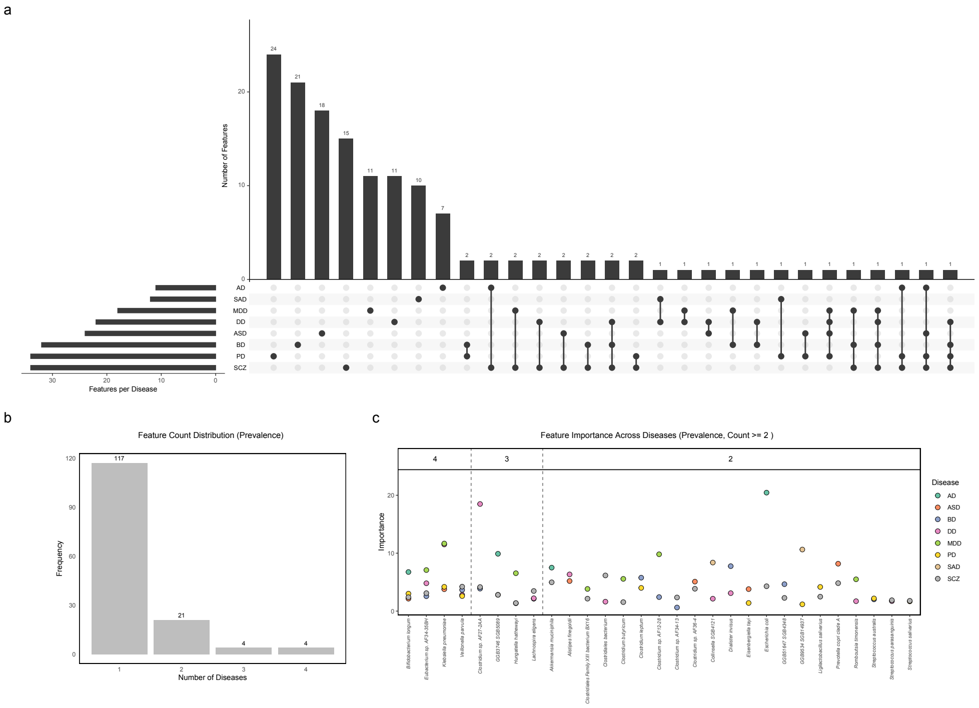

Figure S10. Selected features of disease-specific classifiers based on prevalence data

a) Upset analysis depicting the intersections of features across different disease-specific diagnostic models.

b) Number of feature intersections within each disease-specific classifier.

c) Specific features identified in each disease-specific classifier (n > 2).

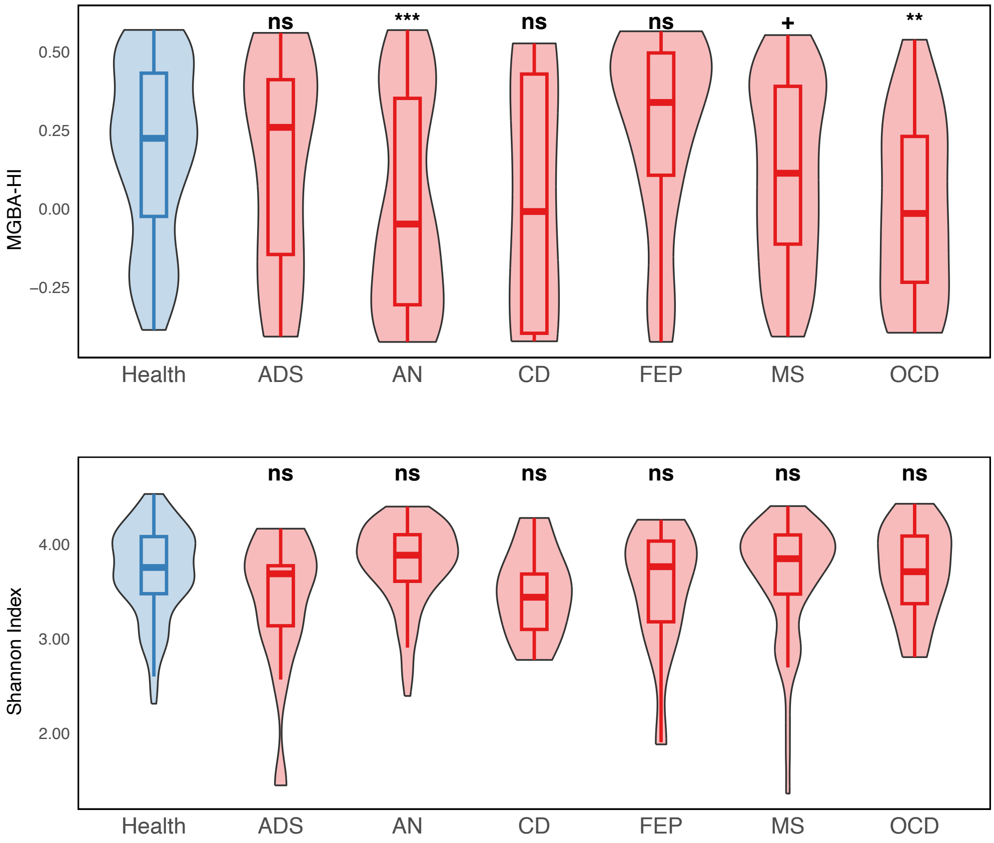

Figure S11. MGBA-HI evaluation in disease-expended dataset

a-b) Comparative distributions of MGBA-HI and Shannon diversity indices between healthy (light blue) and disease (pink) groups in Disease-expended dataset (n = 358). Statistical significance assessed by Wilcoxon rank-sum test.
